# Driving performance and neurocognitive skills of long‐term users of sedating antidepressants

**DOI:** 10.1002/hup.2762

**Published:** 2020-10-01

**Authors:** Nick N.J.J.M. van der Sluiszen, Annemiek Vermeeren, Joke H. van Dijken, Aurora J.A.E. van de Loo, Janet L. Veldstra, Dick de Waard, Joris C. Verster, Karel A. Brookhuis, Johannes G. Ramaekers

**Affiliations:** ^1^ Department of Neuropsychology and Psychopharmacology Faculty of Psychology and Neuroscience Maastricht University Maastricht The Netherlands; ^2^ Department of Clinical and Developmental Neuropsychology University of Groningen Groningen The Netherlands; ^3^ Division Pharmacology Utrecht University Utrecht The Netherlands; ^4^ Institute for Risk Assessment Sciences Utrecht University Utrecht The Netherlands; ^5^ Centre for Human Psychopharmacology Swinburne University Melbourne Australia

**Keywords:** antidepressants, driving performance, long‐term use, neurocognition, on‐the‐road driving

## Abstract

**Objective:**

To assess driving performance and neurocognitive skills of long‐term users of sedating antidepressants, in comparison to healthy controls.

**Methods:**

Thirty‐eight long‐term (>6 months) users of amitriptyline (*n* = 13) and mirtazapine (*n* = 25) were compared to 65 healthy controls. Driving performance was assessed using a 1‐h standardised highway driving test in actual traffic, with road‐tracking error (standard deviation of lateral position [SDLP]) being the primary measure. Secondary measures included neurocognitive tasks related to driving. Performance differences between groups were compared to those of blood alcohol concentrations of 0.5 mg/ml to determine clinical relevance.

**Results:**

Compared to controls, mean increase in SDLP of all antidepressant users was not significant, nor clinically relevant (+0.75 cm, 95% CI: −0.83 cm; +2.33 cm). However, users treated less than 3 years (*n* = 20) did show a significant and clinically relevant increase in SDLP (+2.05 cm). No significant effects were observed on neurocognitive tasks for any user group, although large individual differences were present. Most results from neurocognitive tests were inconclusive, while a few parameters confirmed non‐inferiority for users treated longer than 3 years.

**Conclusion:**

The impairing effects of antidepressant treatment on driving performance and neurocognition mitigate over time following long‐term use of 3 years.

## 1 INTRODUCTION

Despite the availability of non‐sedating antidepressant, older generation antidepressants are still frequently prescribed for the treatment of depression (Arroll et al., 2005; Brauer et al., 2013) and neuropathic pain (Nikolaus & Zeyfang, 2004; Gilron, Baron, & Jensen, 2015). Among this older generation are the tricyclic and tetracyclic antidepressant, such as amitriptyline and mirtazapine (van de Loo et al., 2017). These drugs are known to cause psychomotor‐ and cognitive impairments (Darowski, Chambers, & Chambers, 2009) that interfere with daily activities such as operating a vehicle (Dassanayake, Michie, Carter, & Jones, 2011; Verster & Ramaekers, 2009).

Epidemiological studies showed that tri‐ and tetracyclic antidepressant use is associated with an increased relative risk of 1.4–2.3 of becoming involved in a traffic accident (Bramness, Skurtveit, Neutel, Mørland, & Engeland, 2008; Chang et al., 2013; Leveille et al., 1994; Ray, Fought, & Decker, 1992). Experimental studies in patients confirm that these drugs can produce significant driving impairment after treatment initiation but also indicated that such impairment may decrease over 2 weeks of treatment, possibly due to tolerance (Brunnauer et al., 2008; Veldhuijzen et al., 2006). In line with this, a study found that patients treated chronically with sedating antidepressants (clomipramine or imipramine) showed only minor impairment of psychomotor performance and memory as compared to healthy controls (Gorenstein, De Carvalho, Artes, Moreno, & Marcourakis, 2006). This raises the question whether long‐term use of sedating antidepressants is still associated with clinically relevant impairment of driving.

Experimental studies have systematically assessed the clinical relevance of the effects of antidepressants on driving behaviour by means of comparison to alcohol, given its well documented dose dependent association with crash risk (Blomberg, Peck, Moskowitz, Burns, & Fiorentino, 2009; Borkenstein, Crowther, & Shumate, 1974). These studies focussed on driving performance after single and repeated doses of a range of antidepressants (Ramaekers, 2003). Results showed that tricyclic antidepressants, such as amitriptyline, produce moderate to severe impairment of driving performance equivalent to driving under the influence of a blood alcohol concentration (BAC) of 0.5 mg/ml or more during the first days of treatment as compared to placebo. However, driving impairment was virtually absent after one week of repeated dosing, likely due to tolerance development. For tetracyclic antidepressants, such as mirtazapine, clinically relevant driving impairment was observed at the onset of a nocturnal treatment regimen. This mitigated over 3 weeks of repeated dosing, but never fully disappeared, suggesting that tolerance was incomplete.

Results from experimental driving studies have also been used for classifying fitness to drive of individuals receiving antidepressant treatment. These classification systems (de Gier, Alvarez, Mercier‐Guyon, & Verstraete, 2009; Ravera et al., 2012) use a graded level warning system that expresses drug‐induced impairment in BAC equivalents. The common classifications are: no/minor influence (category 0/I, BAC < 0.5 mg/ml), moderate influence (category II, 0.5 mg/ml ≤ BAC ≤ 0.8 mg/ml), and severe influence (category III, BAC > 0.8 mg/ml). Users of antidepressants that are classified as category III, are advised to not operate a vehicle, given that driving may be impaired for approximately 24 h after intake (Gómez‐Talegón, Fierro, Del Río, & Álvarez, 2011). A limitation of existing drug categorisation systems is the lack of information regarding the effects of long‐term drug usage on driving performance. For example, mirtazapine and amitriptyline are classified as category III drugs because of their acute effects on driving performance. Impairments may however dissipate after prolonged use, in which case classification of these antidepressants as category III may be too conservative for drivers receiving long‐term treatment, limiting their mobility unnecessarily.

The objective of the present study was to evaluate driving performance of long‐term users of category III antidepressants, as compared to that of a group of normative healthy controls. Long‐term usage was defined as longer than 6 months. The secondary objective was to evaluate driving performance separately for those participants who had been using antidepressant for less than 3 years, and those whose use exceeded 3 years. The criterion of 3 years was based on Dutch laws, stating that antidepressant users are unfit to drive when treated for less than 3 years but can request an individual driver fitness evaluation after more than 3 years of stable usage (Ministry of Infrastructure and Water Management, 2000). Driving performance was assessed by a standardised highway driving test in actual traffic and various neurocognitive tests related to driving. The present data were collected as part of a larger study on the long‐term effects of benzodiazepines and antidepressants on driving performance. Data on long‐term benzodiazepine use and driving are published separately (van der Sluiszen et al., 2019).

## 2 METHODS

### 2.1 Design

The study was designed as a multi‐centre trial (Universities of Maastricht, Utrecht and Groningen) in the Netherlands to compare on‐the‐road driving and driving related skills between long‐term (>6 months) users of antidepressants and healthy controls. To explore the potential difference in impairment before and after 3 years of use, antidepressant users were divided into two groups based on duration of treatment, that is, long‐term use less than 3 years (LT3‐) and long‐term use more than 3 years (LT3+).

### 2.2 Participants

Category III antidepressant users were recruited via patient organisations, hospitals, practitioners affiliated with UPPER (Koster, Blom, Philbert, Rump, & Bouvy, 2014) and regional advertisements. Healthy controls were recruited via flyers and advertisements in local newspapers. Participants were informed about the study's goal, procedures and potential hazards. The study was approved by the Medical Ethics Committees of Maastricht University and the Maastricht Academic Hospital and was conducted in agreement with the code of ethics on human experimentation established by the Declaration of Helsinki (1964), amended in Edinburgh (2000), Seoul (2008) and Fortaleza (2013). Written informed consent was obtained from each participant before enrolment. Participants received a financial compensation for their participation in the study.

#### 2.2.1 Antidepressant users

Thirty‐eight long‐term category III antidepressants users were recruited (17 in Maastricht, 14 in Groningen, and 7 in Utrecht). Thirteen used amitriptyline, and 25 used mirtazapine. Initial screening was based on a medical history questionnaire evaluated by research physicians (MDs) responsible for the medical well‐being of participants at each site. The inclusion criteria were: use of a category III antidepressant over a period of at least six months with a frequency of at least two times a week (≈90 days a year), possession of a valid driver's licence for at least 3 years, driving an average of 3000 km per year, normal or corrected to normal vision, body mass index (BMI) between 17 and 35 kg/m^2^. Although Dutch law deems category III antidepressant users who have been treated for less than 3 years unfit to drive, many of them drive a motor vehicle simply because they are unaware of this legal provision and because this provision is not actively enforced by the Dutch government. Participants were excluded if they used concomitant medication classified as International Council on Alcohol, Drugs and Traffic Safety (ICADTS) category III. Concomitant medication classified as ICADTS category 0/I was allowed, whereas ICADTS category II was evaluated by a research physician on individual basis. Additional exclusion criteria were: alcohol use >21 standardised units per week, smoking >20 cigarettes a day, use of illegal drugs in the past 3 months.

Before test days, antidepressant users were requested to take their medication at their usual time of day, that is, in the evening or morning. Their usual dosing regime was established at the screening visit and monitored by self‐report on the practice and test day.

#### 2.2.2 Controls

Sixty‐five controls formed a normative group with comparable age, gender distribution and years of driving experience as the antidepressant users group. Inclusion criteria were: a valid driver's licence for at least 3 years; driving an average of 3000 km per year, normal or corrected to normal vision and BMI between 19 and 29 kg/m^2^. Exclusion criteria were: diagnosed with a neurological‐, psychiatric‐ or sleeping disorder, alcohol use >21 standardised units per week, smoking >10 cigarettes a day and illegal drug use and psychoactive medication (e.g.,: antidepressants, benzodiazepines, anticonvulsants, antihistamines, opioids) in the past 3 months.

### 2.3 On‐the‐road driving test

**FIGURE 1:**
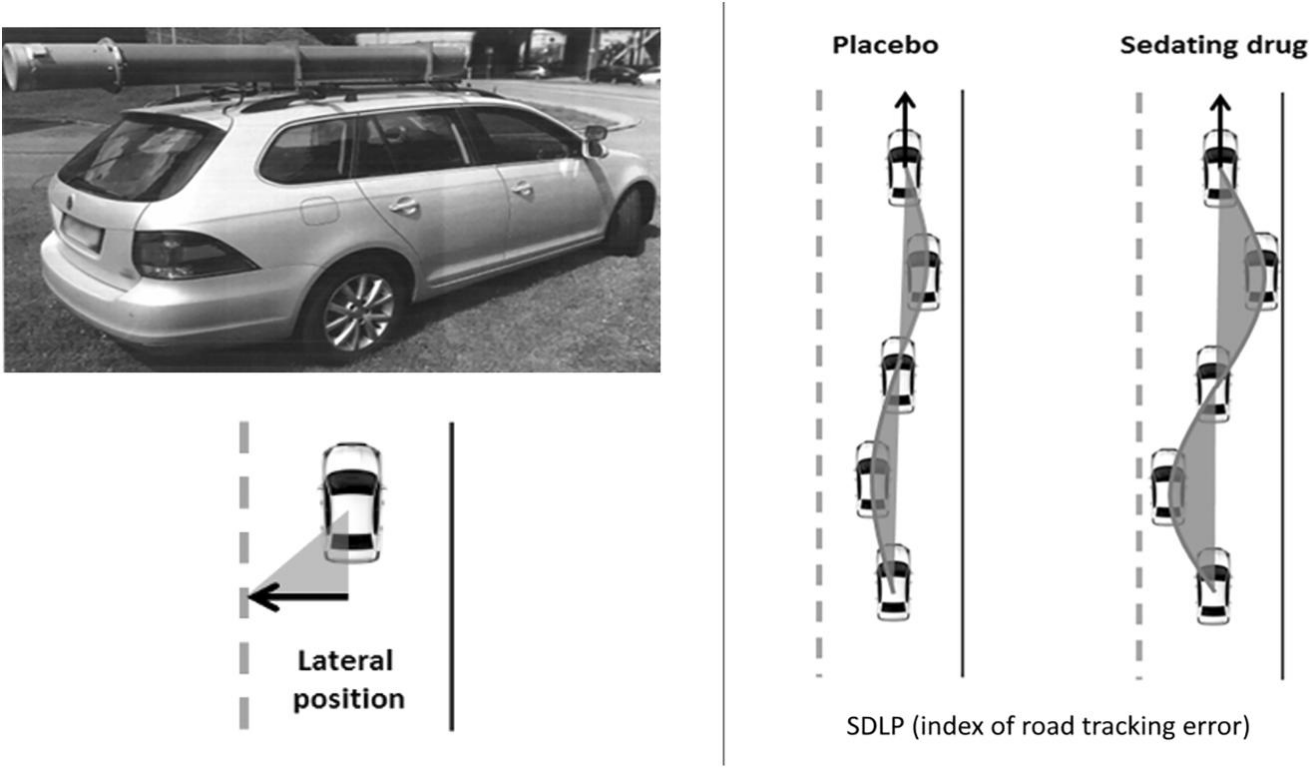
Schematic drawing of the highway driving test. The standard deviation of lateral position (SDLP) is an index of road tracking error or ‘weaving’. Drugs that induce sleepiness or sedation cause loss of vehicle control, leading to increased road tracking error. Figure and description taken with permission from van der Sluiszen et al. (2019)

In the standardised on‐the‐road highway driving test (Figure 1) (O'Hanlon, 1984; Ramaekers, 2017; Verster & Roth, 2011) participants drive a specially instrumented car over a 100 km primary highway circuit (i.e., A2 near Maastricht, A12 near Utrecht, and A28 near Groningen) They are accompanied by a licensed driving instructor having access to dual controls. The participant's task is to maintain a constant speed of 95 km/h and a steady lateral position between the delineated boundaries of the slower right‐hand traffic lane. The vehicle's speed and lateral position relative to the left lane delineation is continuously recorded. These signals are digitally sampled at 4 Hz and pre‐processed off‐line to mark data recorded during overtaking manoeuvres or disturbances caused by roadway or traffic situations. The pre‐processed dataset is used to calculate the mean and variance of lateral position of clean (unmarked) data, for each successive 5‐km segment and, as the square root of pooled variance over all segments, for the test as a whole. The primary outcome variable is the standard deviation of lateral position (SDLP, in cm) which is a measure of road tracking error, or ‘weaving’. SDLP scores of prematurely terminated tests are calculated from the data collected until termination of each ride.

Drug‐induced impairments in the standardised highway driving test have been compared to that of a well‐known benchmark drug (i.e., alcohol) that is known to jeopardise traffic safety and shows a clear exponential dose‐dependent relationship with accident crash risk (Blomberg et al., 2009; Borkenstein et al., 1974). The clinical relevance of performance changes in the highway driving test have previously been determined by establishing the relationship between BAC and SDLP (Louwerens, Gloerich, DeVries, Brookhuis, & O'Hanlon, 1987). A recent meta‐analysis of nine alcohol‐calibration studies revealed that a mean increment in SDLP of 2.5 cm (95% C.I. 2.0–2.9 cm) was observed during the standardised highway driving test at a BAC of 0.5 mg/ml and has been defined as the minimal cut‐off value to present clinical relevance (Jongen et al., 2017). The highway driving test has been used in more than 100 studies and has proven sensitivity to alcohol, antidepressants and many other sedating drugs (Ramaekers, 2017; Roth, Eklov, Drake, & Verster, 2014; Vermeeren, 2004).

### 2.4 Neurocognitive tests

#### 2.4.1 Trailmaking Test

The Trailmaking Test (TMT) is a paper‐and‐pencil test measuring selective and divided attention, as well as executive functions (Reitan, 1958). The test comprises of two parts. In part A, the task of the participant is to connect as fast as possible 25 circles that contain the numbers 1 to 25, by means of connecting the circles in ascending order. In part B, the 25 circles contain letters (A to L) and numbers (1 to 13). Participants are required to connect as fast as possible the 25 circles in an alternately ascending fashion (i.e., 1‐A‐2‐B‐3‐C and so on). The maximum time allowed for part A is 5 min, and for part B it is 6 min. The primary outcome measures for parts A and B is the time (in seconds) needed to complete each task, as measured by a hand‐held stopwatch.

#### 2.4.2 Digit Symbol Substitution Test

The Digit Symbol Substitution Test (DSST) is a paper‐and‐pencil test measuring executive attention and processing speed (Wechsler, 1958). Participants are presented with rows of digits and have to respond by writing the corresponding symbol in a blank space, according to a key presented at the top of the paper. The primary outcome measure is the number of correctly filled‐in symbols in 90 s.

#### 2.4.3 Adaptive Tachistoscopic Traffic Perception Test

The Adaptive Tachistoscopic Traffic Perception Test (ATTPT) of the Vienna Test System assesses visual orientation ability, visual observational ability, speed of perception and skills in obtaining an overview (Schuhfried, 2009). Participants are briefly presented with pictures of traffic situations on a computer screen. After each picture participants are required to indicate what was in the picture, by choosing from five answer options (i.e., cars, cyclists, pedestrians, traffic signs and/or traffic lights). Pictures are presented adaptively, meaning that the difficulty of the pictures is adapted to the abilities of the participant (i.e., participants who perform poorly, receive pictures containing less complex traffic situations; vice versa for participants whom perform well). The primary outcome is the number of correctly identified elements. Time to complete the task is 10 min.

#### 2.4.4 Reaction Test

The Reaction Test (RT) of the Vienna Test System assesses reaction time and motor time in response to simple and complex visual or acoustic signals (Prieler, 2008). Before the start of the test, participants are instructed to lay their index finger on a pressure‐sensitive key (i.e., rest key). During the test, participants are required to press a target key, with their index finger, whenever a target stimulus is presented. After pressing the target key, they must return their index finger immediately to the rest key. By means of using a rest key and target key, it is possible to distinct between reaction time (time between the presentation of the target stimulus and the moment the index finger is removed from the rest key) and motor time (the time between releasing the rest key and pressing the target key). The current experiment uses three versions of the reaction test, namely: S1, in which participants have to respond whenever a yellow circle is shown on screen; S2, in which participants have to respond whenever they hear a tone and S3, in which participants have to respond whenever they see a yellow circle on screen and a hear a tone in combination, all other stimuli combinations are to be ignored. Time to complete all three versions of this task is 10 min. Outcome measures for each test are reaction time and motor time.

#### 2.4.5 Determination Test

The Determination Test (DT) of the Vienna Test System measures reactive stress tolerance, divided attention and mental flexibility (Neuwirth & Benesch, 2007). The test measures the ability to sustain attention over a period of approximately 10 min. Participants are presented with visual stimuli of varying colour and sounds with a different pitch, in a serial order. For each stimulus, a pre‐defined button has to be pressed. The presentation of stimuli is adaptive to the reaction speed of the participant, meaning that the inter‐stimulus‐interval is shortened when participants make correct and fast responses, and is slowed down when participants make mistakes or respond slowly. During the task, participants are presented with the following stimuli and have to press the following corresponding buttons: (a) visual coloured circles (white, yellow, red, green and blue), each presented colour has a matching coloured key on the keyboard; (b) auditory signals (low pitch & high pitch), each auditory signal has its own response key on the keyboard; (c) motor signals (displayed as a white rectangle on the left or right side of the bottom of the screen), each motor signal required the participant to press a left or right foot pedal, depending on the position of the white rectangle on screen. The outcome measure is the average reaction time of all responses made.

#### 2.4.6 Risk‐Taking Test Traffic

The Risk‐Taking Test Traffic (RTTT) measures risk‐taking behaviour in potentially dangerous driving situation (Hergovich, Bognar, Arendasy, & Sommer, 2005). Participants are presented with 24 items (i.e., video clips) that show diverse driving situations, which are described in words before they are shown on‐screen. Each driving situation is shown twice. During the first time, participants observe the entire driving situation. During the second time, participants are required to press a key on the keyboard, indicating the distance from the potential hazard at which the driving manoeuvre that has just been described becomes critical or dangerous (i.e., the point at which the participant would no longer perform the manoeuvre). The first item, of the 24 items serves as a practice item. Time to complete the task is approximately 15 min. The variable ‘willingness to take risk in driving situations’ is measured by obtaining the distance between the moment of a potential hazard, measured in hundreds of a second, and the moment the participant presses the key indicating the potential hazard becomes critical or potentially dangerous. This distance is a measure of subjectively accepted level of risk. Higher scores indicate higher levels of subjectively accepted risk.

#### 2.4.7 Psychomotor Vigilance Test

The Psychomotor Vigilance Test (PVT) is based on a simple visual reaction time test (Dinges & Powell, 1985). It measures the ability to sustain attention over a period of approximately 10 min. Participants are required to respond to a visual stimulus presented at a variable interval (2–10 s) by pressing a button with the dominant hand. The visual stimulus is the presentation of a counter that starts running from 0 to 60 s at 1‐ms intervals. Participants are required to respond to this visual counter as soon as they perceive it on screen by pressing the corresponding button. If a response is made the counter stops, stays on screen for 500 ms as visual feedback for the participant, and disappears. During this period, a variable interval is presented and afterwards the next counter appears on screen. This cycle repeats until 100 stimuli have been presented on screen. If a response has not been made within 60 s, the clock resets and the counter restarts. Primary outcome measures are mean response speed and number of lapses (defined as responses with RT ≥ 500 ms) (Basner & Dinges, 2011). Performance on the PVT has been calibrated for dose‐effects of alcohol (Jongen, Vuurman, Ramaekers, & Vermeeren, 2014).

### 2.5 Questionnaires

#### 2.5.1 Beck's Depression Inventory

The Beck's Depression Inventory (BDI; Beck, Steer, & Carbin, 1988) is a 21‐item self‐report questionnaire measuring depression related symptomology. Answer options for each question range from 0 to 3. The obtained total score for the BDI serves as an indicator for the presence of depression related symptoms, ranging from 0 to 63. Higher scores indicate the presence of more symptoms of depression.

#### 2.5.2 State–Trait Anxiety Index—Trait

The State–Trait Anxiety Index—Trait (STAI‐T; Spielberger, Gorsuch, & Lushene, 1970) is the Trait dimension of the 40‐item self‐reported STAI questionnaire. The STAI‐T contains 20 questions that measure trait anxiety (i.e., how individuals feel in general). Answer options for each questions range from 1 to 4, with total scores ranging from 20 to 80. Higher total scores indicate more anxiety related symptoms.

#### 2.5.3 Pittsburgh Sleep Quality Index

The Pittsburgh Sleep Quality Index (PSQI; Buysse, Reynolds, Monk, Berman, & Kupfer, 1989) is a self‐report questionnaire that assesses the quality and patterns of sleep over the last month, by rating the following seven domains: subjective sleep quality, sleep latency, sleep duration, habitual sleep efficiency, sleep disturbance, use of medication and daytime disturbance. A summary score ranging from 0 to 21 can be derived, with higher scores indicating poorer sleep quality. A summary score ≥5 indicates a poor sleeper.

#### 2.5.4 Groningen Sleep Quality Scale

The Groningen Sleep Quality Scale (GSQS; van der Meulen; Wijnberg, Hollander, De Diana, & van den Hoofdakker, 1980) is a 14‐item self‐report scale that assess subjective quality of sleep during the previous night. Summary scores range from 0 to 14, with higher scores indicating poorer sleep quality. A total score ≥6 indicates disturbed sleep.

### 2.6 Procedure

All participants completed a practice session and a test session on separate days, with an interval of one week between both days. Participants started at 8:30 h, 10:30 h or 12:30 h based on individual convenience, but the starting time was constant on practice and test days. On arrival at practice and test days, participants were screened for use of alcohol (breath test), illegal drugs (urine test) and caffeine (self‐report). On Day 1 (practice day), participants filled out three questionnaires (BDI, PSQI and STAI‐T) and were familiarised with the test procedures. Participants were individually trained to perform the driving test and all neurocognitive tests. On Day 2 (test day), participants first completed the GSQS, followed by the first part of the neurocognitive test battery (TMT, DSST, ATTPT, RT and DT). After a 15‐min break, participants completed the second part of the test battery (RTTT and PVT). Finally, participants were transported to the start of the highway to start the 1‐h driving test. In total, the test day lasted approximately 4:00 h (Table 1).

**TABLE 1:** Overview testing day

Time (h:min)	Event
+0:00	Inclusion check
	Alcohol, drugs, precepts, questionnaires
+0:20	Neurocognitive tests—Part I
	TMT; DSST; ATTPT; RT‐S1,‐S2,‐S3; DT
+1:20	Break
	Standardised meal and refreshment
+1:35	Neurocognitive tests—Part II
	RTTT; PVT
+2:20	Transport to highway
+2:30	Start highway driving test
+3:30	End highway driving test, transport to lab
+4:00	End testing day

*Note*: Time (in hours) is displayed relative from start.

Abbreviations: ATTPT, Adaptive Tachistoscopic Traffic Perception Test; DSST, Digit Symbol Substitution Test; DT, Determination Test; PVT, Psychomotor Vigilance Test; RT, Reaction Test; RTTT, Risk Taking Test Traffic; TMT, Trail Making Test.

### 2.7 Statistical analysis

Statistical power to detect a clinically relevant mean difference in SDLP of 2.5 cm between antidepressant users and controls was as follows: all antidepressant users versus controls, *β* = 0.88; antidepressants LT3−users versus controls, *β* = 0.73; antidepressant LT3+ users versus controls, *β* = 0.70. Assumptions for these power calculations are: an alpha of 0.05 and a between‐subject SD of 4.3 cm (Jongen et al., 2017).

Matching variables (age, gender and driving experience) were included as covariates in an ANCOVA model. If none of the matching variables showed a significant influence on group‐comparisons with SDLP or a neurocognitive parameter, antidepressant users performance was compared to that of the entire group of controls (*n* = 65). Alternatively, if one (or more) matched variables did show a significant influence on a between‐group comparison, only individually matched controls were included. The determination of the influence of matching variables was performed for SDLP and each neurocognitive parameter separately.

**FIGURE 2:**
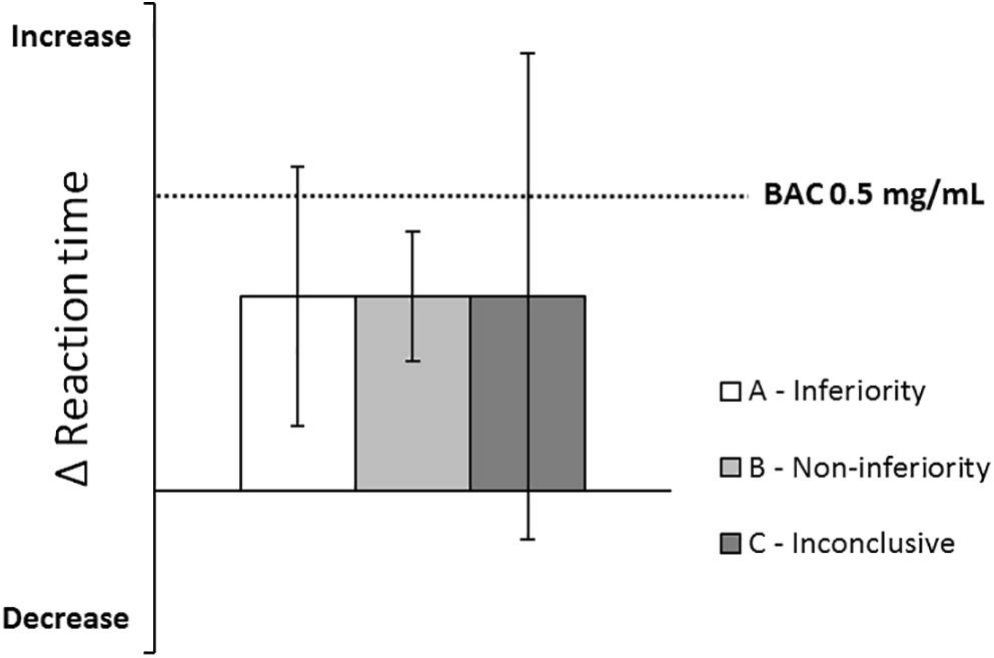
Hypothetical example of the qualification of clinical relevance of performance differences between antidepressant users and controls. The dotted line indicates the change in performance after alcohol intake (relative to placebo). A (drug‐induced) change in performance will be classified as inferior when the 95% CI includes the alcohol criterion but not zero (A—inferiority). Non‐inferiority is concluded when the 95% CI does not include the alcohol criterion (B—non‐inferiority). If the 95% CI includes the alcohol criterion as well as zero, the qualification of clinical relevance is undecided (C—inconclusive). Figure and description taken with permission from van der Sluiszen et al. (2019)

**FIGURE 3:**
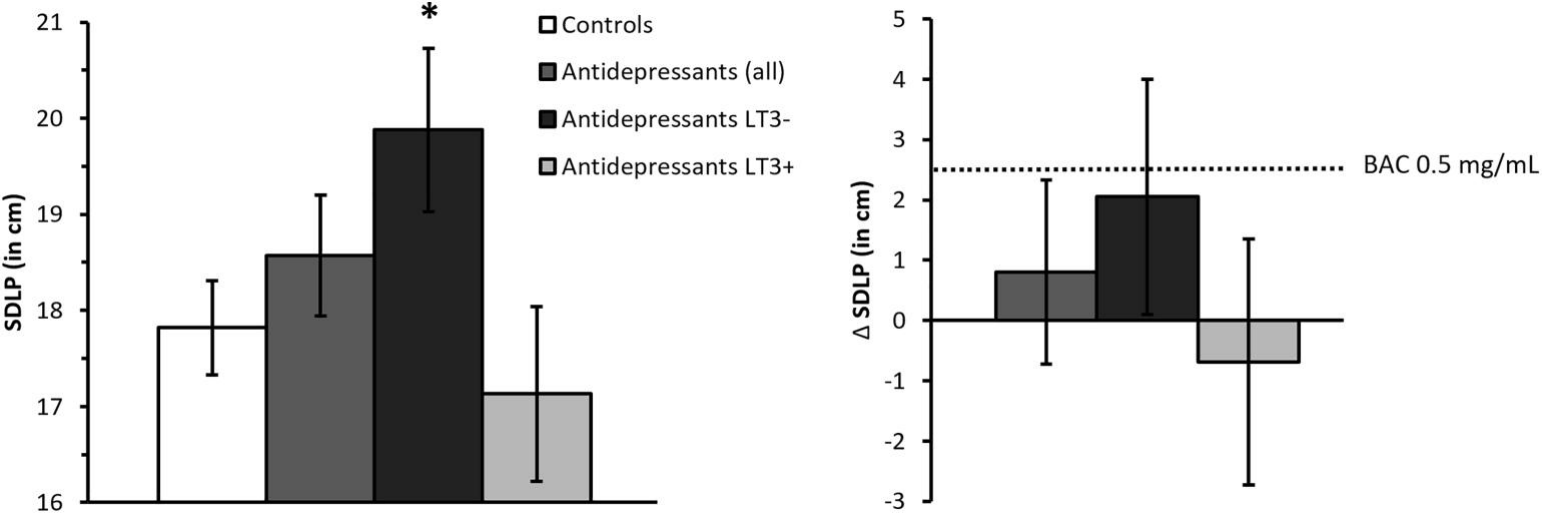
Left: Mean (±SE) SDLP for controls and antidepressant user groups. Right: mean (95% CI) differences in SDLP between antidepressant user groups and controls. The dotted line indicates the change in performance after alcohol intake (relative to placebo). Symbols above bars indicate significant difference from controls, *p* < 0.05. BAC, blood alcohol concentration; LT3−, users treated less than 3 years; LT3+, users treated longer than 3 years; SDLP, standard deviation of lateral position

Next, non‐inferiority analyses were used to determine whether the 95% confidence interval (CI) of performance differences between antidepressant users and controls exceeded the criterion level of clinical relevance, that is, an equivalent performance change as seen at a BAC of 0.5 mg/ml. When evaluating the 95% CI of differences between groups, three interpretations are possible (Figure 2). Antidepressant users' performance was considered not impaired (i.e., non‐inferior) when the upper limit of the 95% CI of the difference from controls was below the alcohol criterion for impairment. Their performance was considered impaired (i.e., inferior) when the lower limit of the 95% CI of the difference from controls was above zero and the upper limit exceeded the alcohol criterion for impairment. When the 95% CI of the difference from controls included both zero and the alcohol criterion for impairment, the results were considered inconclusive. The non‐inferiority limit for the on‐the‐road driving test (Figure 3) was obtained from Jongen et al. (2017).

Clinical relevance of impairment of neurocognitive performance was also based on direct comparison with the impairing effects of alcohol obtained at a BAC of 0.5 mg/ml. In a separate study (Verster et al., 2016) an alcohol‐calibration was performed to determine which neurocognitive parameters were able to detect impairment at a BAC of 0.5 mg/ml. Results of the calibration study showed that the only parameters sensitive for the impairing effects of alcohol were: TMT‐A, DSST, RT‐S1, RT‐S2, RT‐S3, DT and PVT. Consequently, these are the only parameters that provided non‐inferiority limits for the present study. The clinical relevance of results will only be discussed for these parameters.

All statistical analyses were conducted by using the IBM Statistical Package for the Social Sciences for Windows (version 24; IBM Corp.). Power calculations were performed using G*Power version 3.1 (Faul, Erdfelder, Lang, & Buchner, 2007).

## 3 RESULTS

### 3.1 Group characteristics

Table 2 shows a demographic summary of the antidepressant user group (*n* = 38), user subgroups (LT3−, *n* = 20; LT3+, *n* = 18) and the control group (*n* = 65). As expected, significant differences between antidepressant users and controls were found for the BDI (*F*_1,101_ = 42.72, *p* < 0.01), STAI (*F*_1,101_ = 42.07, *p* < 0.01), PSQI (*F*_1,101_ = 23.72, *p* < 0.01), GSQS (*F*_1,101_ = 11.78, *p* < 0.01). Antidepressant users showed more symptomology related to anxiety, depression and sleep problems. Furthermore, none of the participants smoked before arrival at the test site, nor during the test day.

**TABLE 2:** Demographic data of antidepressant users and control groups

	Antidepressant users (all) (*n* = 38)	Antidepressant users LT3‐ (*n* = 20)	Antidepressant users LT3+ (*n* = 18)	Healthy Controls (*n* = 65)
Gender (male/Female)	23/15	10/10	13/5	37/28
Age (years)	54.0 ± 12.7	51.3 ± 12.6	57.1 ± 12.4	57.9 ± 10.5
Body Mass index	27.2 ± 3.7	26.6 ± 3.0	27.9 ± 4.3	25.5 ± 3.0
Driving experience (km/year)	11,605 ± 7689	9740 ± 5162	13,678 ± 9499	13,659 ± 9477
Depression symptoms (BDI)	9.4 ± 7.7	11.5 ± 7.6	7.1 ± 7.3	2.5 ± 2.8
Anxiety symptoms (STAI‐T)	38.6 ± 12.7	43.6 ± 13.1	33.0 ± 9.8	27.1 ± 5.2
Sleep problems (PSQI)	5.6 ± 3.3	6.7 ± 3.6	4.3 ± 2.6	2.8 ± 2.4
Sleep complaints pre‐testing (GSQS)	3.1 ± 3.1	3.6 ± 2.8	2.5 ± 3.3	1.4 ± 1.8

*Note*: The mean (±SD) of variables are reported.

Abbreviations: BDI, Beck's Depression Inventory; GSQS, Groningen Sleep Quality Scale; LT3−, users treated less than 3 years; LT3+, users treated longer than 3 years; PSQI, Pittsburgh Sleep Quality Index; STAI‐T, State‐Trait Anxiety Index—Trait dimension.

Table 3 shows the use of antidepressant and psycho‐active co‐medication for the long‐term users (sub)group. They took their medication at least once a day. The majority of long‐term users received their antidepressant for the treatment of depressive symptomatology, followed by neuropathic pain treatment. None of the long‐term users received antidepressant treatment for sleep related complaints. The most frequently reported co‐medication were second generation antidepressants (SSRIs/SNRIs), which have minor effects on driving performance (category I). In addition, three users of category III antidepressants reported usage of category II co‐medication.

**TABLE 3:** Psycho‐active medication and daily doses (in mg) used per antidepressant users subgroup

	Antidepressant users LT3‐ (*n* = 20)	*M* ± *SD*	*N*	Antidepressant users LT3+ (*n* = 18)	*M* ± *SD*	N
Cat. III	Amitriptyline, *q.d., nocte*	32.5 ± 29.0	4	Amitriptyline, *q.d., nocte*	63.3 ± 52.7	9
	Mirtazapine, *q.d., nocte*	22.0 ± 12.7	16	Mirtazapine, *q.d., nocte*	26.7 ± 12.5	9
Cat. II	Olanzapine, *q.d*.	2.5	1	Nortriptyline, *q.d*.	100.0	1
				Quetiapine, *b.i.d*.	100.0	1
Cat. I	(es)citalopram, *q.d*.	15.0 ± 7.1	2	Escitalopram, *q.d*.	10.0	1
	Sertraline, *q.d*.	50.0	1	Venlafaxine, *q.d*.	168.8 ± 185.6	2
	Venlafaxine, *q.d*.	150.0	3			

Abbreviations: b.i.d., twice a day; Cat. I, category I co‐medication; Cat. II, category II co‐medication; Cat. III, category III medication; LT3−, users treated less than 3 years; LT3+, users treated longer than 3 years; nocte, bedtime administration; q.d., once a day.

### 3.2 Matching of controls

Analyses showed no significant effect of age, gender or driving experience in the ANCOVA for either SDLP, ATTPT, RTTT and PVT mean reaction time. For these parameters, the entire control group was used as a reference for comparison with the long‐term users groups. For the remaining parameters, matched healthy controls were used for each long‐term users (sub)group.

### 3.3 Highway driving test

Data of the highway driving test were missing for one person in the control group due to problems with the recording system. None of the individual driving tests were prematurely terminated.

Mean (±SE) scores for SDLP of both groups are shown in Figure 3. Mean SDLP of all antidepressant users did not differ significantly from controls (*F*_1,100_ = 0.89, *p* = 0.35). The upper limit of the 95% CI of the difference between both groups (+0.75 cm, 95% CI: −0.83 cm; +2.33 cm) did not exceed the +2.5 cm criterion, indicating non‐inferiority. Further analysis showed a significant difference between LT3− users and controls (*F*_1,82_ = 4.40, *p* = 0.04), but not between LT3+ users and controls (*F*_1,80_ = 0.46, *p* = 0.50). Mean (95% CI) difference in SDLP between LT3− users and controls was +2.05 cm (+0.11 cm; +4.00 cm) and −0.69 cm (−2.74 cm; +1.35 cm) for LT3+ users. Non‐inferiority testing revealed that only for LT3− users, the lower and upper limit of the mean difference in overall SDLP exceed zero and the +2.5 criterion respectively, indicating clinically relevant impairment.

### 3.4 Neurocognitive performance

Table 4 shows the mean (±SE) of all performance parameters for each antidepressant users (sub)group and healthy controls. Comparisons between antidepressant users and controls showed no significant performance difference between both groups. Table 5 shows an overview of the 95% CI of mean changes between antidepressant users and (matched) controls on alcohol sensitive parameters only, including inferiority limits and analyses. The 95% CI of mean changes of all alcohol sensitive parameters included zero and exceeded the BAC 0.5 mg/ml criterion, indicating inconclusive results.

**TABLE 4:** Mean (±SE) of all performance parameters for each antidepressant users group and the (matched) healthy control group

All controls	Antidepressant users (all), *N* = 38	Antidepressant users LT3−, *N* = 20	Antidepressant users LT3+, *N* = 18	All Controls, N = 65
SDLP (in cm)	18.5 ± 0.6	19.9 ± 0.9*	17.1 ± 0.9	17.8 ± 0.5
ATTPT (number correct)	95.1 ± 1.9	94.3 ± 2.7	96.1 ± 2.9	98.0 ± 1.5
RTTT	8.0 ± 0.2	7.8 ± 0.3	8.1 ± 0.4	7.9 ± 0.2
PVT mean RT (ms)	295 ± 6	306 ± 8	281 ± 8	289 ± 5

Matched controls	Antidepressant users (all), *N* = 38	Matched controls, *N* = 30	Antidepressant users LT3−, *N* = 20	Matched controls, *N* = 14	Antidepressant users LT3+, *N* = 18	Matched controls, *N* = 16
TMT‐A (sec)	30.2 ± 1.8	26.8 ± 2.0	31.7 ± 2.5	24.4 ± 3.0	28.4 ± 2.5	28.9 ± 2.6
TMT‐B (sec)	62.1 ± 3.8	56.3 ± 4.3	57.5 ± 3.6	50.0 ± 4.3	67.3 ± 6.8	61.9 ± 7.2
DSST (number correct)	56.6 ± 1.7	57.5 ± 1.9	57.5 ± 2.5	59.8 ± 3.0	55.6 ± 2.3	55.6 ± 2.5
RT‐S1						
Reaction time (ms)	270 ± 8	272 ± 9	272 ± 9	257 ± 11	269 ± 13	286 ± 13
Motor time (ms)	162 ± 10	151 ± 7	168 ± 14	151 ± 9	156 ± 14	151 ± 7
RT‐S2						
Reaction time (ms)	230 ± 7	234 ± 8	239 ± 9	217 ± 11	221 ± 11	250 ± 12
Motor time (ms)	151 ± 7	150 ± 8	159 ± 9	137 ± 11	142 ± 11	161 ± 12
RT‐S3						
Reaction time (ms)	440 ± 14	441 ± 16	458 ± 20	408 ± 24	419 ± 19	469 ± 21
Motor time (ms)	175 ± 9	181 ± 10	178 ± 10	165 ± 12	173 ± 14	195 ± 15
DT reaction time (ms)	850 ± 18	872 ± 20	829 ± 24	836 ± 29	874 ± 26	904 ± 28
PVT lapses (number)	2.8 ± 0.5	2.0 ± 0.6	3.8 ± 0.7	1.5 ± 0.9	1.7 ± 0.7	2.5 ± 0.7

Abbreviations: ATTPT, Adaptive Tachistoscopic Traffic Perception Test; DSST, Digit Symbol Substitution Test; DT, Determination Test; LT3−, users treated less than 3 years; LT3+, users treated longer than 3 years; PVT, Psychomotor Vigilance Test; RT, Reaction Test; RTTT, Risk Taking Test Traffic; SDLP, standard deviation of lateral position; TMT, Trail Making Test.

*Indicates significant difference from (matched) control group.

**TABLE 5:** Mean (95% CI) differences in alcohol calibrated neurocognitive parameters between antidepressant users and controls, and non‐inferiority analysis

	Non‐inferiority limit	Antidepressant users (all) *N* = 38		Antidepressant users LT3− *N* = 20		Antidepressant users LT3+ *N* = 18	
TMT‐A (sec)	+2.72	+3.4 (−2.0; +8.7)	*Inc*	+7.4 (−0.7; +15.4)	*Inc*	−0.5 (−7.9; +6.9)	*Inc*
DSST (number correct)	−1.38	−0.9 (−6.0; +4.2)	*Inc*	−2.3 (−10.2; +5.6)	*Inc*	+0.1 (−6.9; +7.0)	*Inc*
RT‐S1							
Reaction time (ms)	+10.35	−1.9 (−25.1; +21.3)	*Inc*	+14.6 (−13.6; +42.9)	*Inc*	−16.7 (−54.2; +20.9)	*Inc*
RT‐S2							
Reaction time (ms)	+7.83	−4.0 (−26.2; +18.3)	*Inc*	+22.6 (−6.9; +52.0)	*Inc*	−29.3 (−62.1; +3.5)	*n‐Inf*
RT‐S3							
Reaction time (ms)	+23.82	−1.2 (−44.4; +42.1)	*Inc*	+49.7 (−14.3; +113.8)	*Inc*	−50.1 (−107.6; +7.4)	*n‐Inf*
DT							
Reaction time (ms)	−9.14	−22.1 (−76.3; +32.2)	*Inc*	−7.4 (−84.5; +69.7)	*Inc*	−29.9 (−106.8; +47.1)	*Inc*
PVT							
Mean (ms)	+19.36	+5.6 (−9.6; +20.8)	*Inc*	+17.4 (−0.6; +35.4)	*Inc*	−7.6 (−25.4; +10.3)	*n‐Inf*
Lapses (number)	+1.71	+0.8 (−0.8; +2.3)	*Inc*	+2.3 (−0.1; +4.6)	*Inc*	−0.8 (−2.7; +1.2)	*n‐Inf*

Abbreviations: DSST, Digit Symbol Substitution Test; DT, Determination Test; Inc, Inconclusive; n‐Inf, non‐inferior; PVT, Psychomotor Vigilance Test; RT, Reaction Test; TMT, Trail Making Test.

Subsequent analyses based on treatment duration showed no significant performance difference between the normative control group and the LT3− or LT3+ user groups, respectively. Similar to the results for the group as a whole, non‐inferiority analysis of alcohol sensitive parameters showed that the 95% CIs of the difference between LT3− users and controls on all alcohol sensitive parameters included zero and the alcohol criterion indicating inconclusive results. For the LT3+ users subgroup, non‐inferiority was observed for the parameters of the RT‐S2, RT‐S3 and the PVT (mean RT + Lapses).

## 4 DISCUSSION

The current study compared the driving performance of long‐term users of sedating antidepressants to that of a normative control group consisting of healthy participants. The goal was to evaluate whether the classification of the investigated antidepressants in category III may be too conservative for drivers who use their medication for a prolonged time. Overall, mean SDLP of long‐term antidepressant users did not differ significantly from the control group. Significant increments in SDLP were found, however, for antidepressant users who had been treated for less than 3 years, but not for antidepressant users treated for longer than 3 years. Furthermore, antidepressant users showed no significant differences in neurocognitive performance in comparison to controls, although individual variations were large as evidenced by wide 95% CIs around mean differences.

The clinical relevance in performance between antidepressant users and controls was determined by comparison to a threshold based on the influence of a BAC of 0.5 mg/ml. In the present study, when looking at the whole group of antidepressants users, the mean increase in SDLP was 0.75 cm in comparison to healthy controls. The 95% CI of this mean difference included zero and did not include the alcohol criterion. This indicates that performance of antidepressant users during the on‐the‐road driving test is considered non‐inferior for the group as a whole, given that the level of impairment associated with the legal limit of alcohol in traffic was not reached.

However, clinically relevant driving impairment was found in individuals who had been using antidepressants for less than 3 years. The mean (95% CI) difference in SDLP between controls and LT3− users was +2.05 cm (+0.11 cm; +4.00 cm) which includes the BAC 0.5 mg/ml criterion of clinical relevance. For LT3+ users the mean and 95% CI remained below the alcohol criterion. This suggests mitigation of driving‐related impairment over time, which corresponds with a decreasing accident risk found in epidemiological studies following long‐term antidepressant treatment (Barbone, McMahon, & Davey, 1998; Rapoport et al., 2011). Possible factors that may contribute to this mitigation are physiological tolerance, improvements in clinical symptomatology (van der Sluiszen et al., 2017) and behavioural tolerance (i.e., learning to minimise the unwanted drug effects on performance by means of cognitive‐ or behavioural adaptation). Physiological tolerance to the sedating effects of older generation antidepressant may be attributed to desensitisation of central H_1_‐receptors, which mainly cause these sedative effects (Ramaekers, 2003). Previous research shows that the impairing effects of first generation antihistamines mitigates after repeated administration (Theunissen, Vermeeren, & Ramaekers, 2006) but may not fully disappear (Verster & Volkerts, 2004).

Most neurocognitive tests used in the study measure skills related to driving performance (Kay & Logan, 2011). The performance of antidepressant users on these neurocognitive tasks showed no significant differences when compared to normative controls. However, the 95% CIs of the mean differences in performance between users and controls on all neurocognitive task also included the alcohol criterion, suggesting large inter‐individual variations. Mean differences for these parameters should therefore be regarded as inconclusive in terms of clinical relevance. Furthermore, for participants treated longer than 3 years non‐inferiority was observed for four neurocognitive tests. This supports the notion that treatment duration plays a role in the observed impairment in long‐term treated antidepressant users.

The present study may hold several limitations. First, the study employed an observational design in which individuals were included into the study based upon their category III antidepressant usage and gave self‐reported reasons (such as depression or pain) for their prescription. As such, the exact underlying clinical indication remained unknown. This lack of information makes direct comparison with prior research about long‐term antidepressant treatment difficult, given the possibility of disorder heterogeneity. Nonetheless, the present study primarily aimed to include a representative group of drivers with long‐term category III antidepressant treatment. Second, antidepressant users in the present study showed differences in daily dosages and reported co‐medication. Although such factors limit the interpretation of underlying mechanisms, it does reflect the characteristics of the population of long‐term antidepressant users who operate a vehicle. Third, there may be a selection bias, in so far as probably only antidepressant users who estimated themselves as fit‐to‐drive volunteered for the study. However, these individuals may be representative for the target population, that is, long‐term users who are active car drivers. Individuals who do not feel fit‐to‐drive are less likely to drive in real‐life. Fourth, the division of treatment duration into subgroups was based on a practical and legislative measure as adopted in the Netherlands. Nonetheless, future studies could explore the time needed to build op tolerance to the impairing effects of daily antidepressant usage and handle treatment duration as a continues variable over time. Fifth, the current study did not include the collection of blood samples to monitor drug levels. Monitoring driving related impairment as a function of drug levels and/or treatment duration may indicate a time‐point at which the development of tolerance mitigates the magnitude of performance impairment below the level of clinical relevant impairment (van der Sluiszen, Vermeeren, Jongen, Vinckenbosch, & Ramaekers, 2017).

In summary, antidepressant users who were treated for less than 3 years showed clinically relevant impairment during the on‐the‐road driving test, but this was absent in the subgroup of users treated for longer than 3 years. The lack of clinically relevant impairment in antidepressant users treated longer than 3 years was further supported by results from neurocognitive tests, although most outcomes of the neurocognitive tests remained inconclusive. These findings support the idea that duration of treatment can be taken into account when evaluating the impact of long‐term medication usage on individual drivers. The implication would be that classification systems grading the effects of drugs on driving, should allow for differential classification of drug effects on driving based on treatment duration.

## CONFLICT OF INTEREST

J.C. Verster has received grants from Janssen, Nutricia, Red Bull, Sequential, Takeda, and acted as a consultant/advisor for 82Labs, Canadian Beverage Association, Centraal Bureau Drogisterij bedrijven, Clinilabs, Coleman Frost, Danone, Deenox, Eisai, Janssen, Jazz, Purdue, Red Bull, Sanofi‐Aventis, Sen‐Jam Pharmaceutical, Sepracor, Takeda, Transcept, Trimbos Institute, Vital Bevrages and ZBiotics. A. Vermeeren and J.G. Ramaekers have received funding over the last 4 years from pharmaceutical companies (Eisai, Jazz, Merck and Transcept).
